# Improved high-resolution pediatric vascular cardiovascular magnetic resonance with gadofosveset-enhanced 3D respiratory navigated, inversion recovery prepared gradient echo readout imaging compared to 3D balanced steady-state free precession readout imaging

**DOI:** 10.1186/s12968-016-0296-4

**Published:** 2016-11-02

**Authors:** Animesh Tandon, Sassan Hashemi, W. James Parks, Michael S. Kelleman, Denver Sallee, Timothy C. Slesnick

**Affiliations:** 1 Departments of Pediatrics, Radiology, and Biomedical Engineering, University of Texas Southwestern Medical School, Dallas, TX USA; 2 Children’s Medical Center Dallas, Dallas, TX USA; 3 Children’s Healthcare of Atlanta, Atlanta, GA USA; 4 Emory University School of Medicine, Atlanta, GA USA

**Keywords:** Pediatric, Cardiovascular magnetic resonance, Coronary artery imaging, Gadofosveset trisodium

## Abstract

**Background:**

Improved delineation of vascular structures is a common indication for cardiovascular magnetic resonance (CMR) in children and requires high spatial resolution. Currently, pre-contrast 3D, respiratory navigated, T2-prepared, fat saturated imaging with a bSSFP readout (3D bSSFP) is commonly used; however, these images can be limited by blood pool inhomogeneity and exaggeration of metal artifact. We compared image quality of pediatric vasculature obtained using standard 3D bSSFP to 3D, respiratory navigated, inversion recovery prepared imaging with a gradient echo readout (3D IR GRE) performed after administration of gadofosveset trisodium (GT), a blood pool contrast agent.

**Methods:**

For both sequences, VCG triggering was used with acquisition during a quiescent period of the cardiac cycle. 3D bSSFP imaging was performed pre-contrast, and 3D IR GRE imaging was performed 5 min after GT administration. We devised a vascular imaging quality score (VIQS) with subscores for coronary arteries, pulmonary arteries and veins, blood pool homogeneity, and metal artifact. Scoring was performed on axial reconstructions of isotropic datasets by two independent readers and differences were adjudicated. Signal- and contrast-to-noise (SNR and CNR) calculations were performed on each dataset.

**Results:**

Thirty-five patients had both 3D bSSFP and 3D IR GRE imaging performed. 3D IR GRE imaging showed improved overall vascular imaging compared to 3D bSSFP when comparing all-patient VIQS scores (*n* = 35, median 14 (IQR 11–15), vs 6 (4–10), *p* < 0.0001), and when analyzing the subset of patients with intrathoracic metal (*n* = 17, 16 (14–17) vs. 5 (2–9), *p* < 0.0001). 3D IR GRE showed significantly improved VIQS subscores for imaging the RCA, pulmonary arteries, pulmonary veins, and blood pool homogeneity. In addition, 3D IR GRE imaging showed reduced variability in both all-patient and metal VIQS scores compared to 3D bSSFP (*p* < 0.05). SNR and CNR were higher with 3D IR GRE in the left ventricle and left atrium, but not the pulmonary arteries.

**Conclusions:**

Respiratory navigated 3D IR GRE imaging after GT administration provides improved vascular CMR in pediatric patients compared to pre-contrast 3D bSSFP imaging, as well as improved imaging in patients with intrathoracic metal. It is an excellent alternative in this challenging patient population when high spatial resolution vascular imaging is needed.

## Background

The use of cardiovascular magnetic resonance (CMR) in the pediatric population continues to increase, aided by advancements in techniques that accommodate higher heart rates and shorter scanning time. CMR spatial resolution has improved and now approaches that of computed tomography (CT). Improved delineation of vascular structures, including coronary arteries and pulmonary veins, is a common indication for CMR imaging in children. However, this can be challenging given the need for high spatial resolution and the small size of the structures to be imaged.

Currently, pre-contrast 3D, respiratory navigated, T2 prepared, fat saturated imaging with a balanced steady-state free precession readout (3D bSSFP) is commonly used for imaging of pediatric vascular structures [1, 2]. Images generated by the 3D bSSFP technique, however, often demonstrate blood pool inhomogeneity and exaggerate metal artifacts, which is especially challenging in the congenital heart disease population. Imaging large fields of view using the 3D bSSFP technique also requires prolonged scan times due to navigator inefficiency [3]. The development and FDA approval of a protein-bound MR contrast agent, gadofosveset trisodium (GT), has allowed fundamental changes in the high spatial resolution, 3D pulse sequences. Protein binding allows for a longer duration of contrast effects, so that imaging during an equilibrium phase achieves minimal to no change in the degree of T1 shortening over the scan duration. A 3D, respiratory-navigated, inversion recovery prepared sequence with a gradient echo readout (3D IR GRE) leverages this prolonged equilibrium phase for vascular imaging.

Previous studies have compared MR angiography (MRA) with gadofosveset trisodium to traditional gadobenate dimeglumine contrast for coronary arteries in adults at 3.0 T [4]; coronary artery imaging with gadofosveset trisodium at 3.0 T compared to pre-contrast 3D SSFP imaging at 1.5 T [1]; and for other angiography of carotid [5], renal [6, 7], and iliofemoral arteries [6]. The use of gadofosveset trisodium has shown advantages in each of these situations. Some adult studies of gadofosveset vs. gadobenate show no advantage of gadofosveset, as long as the imaging is performed quickly after contrast administration [8]. Studies in adults with congenital heart disease have shown advantages of the use of gadofoseveset [9]. And, though a recent study shows that in adults 3D inversion recovery bSSFP shows improved image quality as compared to 3D IR GRE, our experience at our institution has shown improved imaging with 3D IR GRE [10]. Studies in the pediatric population are limited, showing improved pediatric vascular imaging with gadofosveset and combined respiratory and cardiac triggering compared to without triggering [3], and improved pediatric vascular imaging with gadofosveset and steady-state MRA compared to first-pass MRA [11]. We compared image quality of pediatric vasculature obtained using the standard 3D bSSFP technique to that obtained with 3D IR GRE imaging performed after administration of GT, including patients with repaired congenital heart disease.

## Methods

### Study population

The current study represents a retrospective review of all pediatric patients at our institution who underwent CMR using GT with both pre-contrast 3D bSSFP and post-contrast 3D IR GRE imaging during the same examination. Our institution began using GT for clinical CMR evaluations in August 2013, and the present study included patients from August, 2013 to June, 2014. Patients were excluded if either the 3D bSSFP or 3D IR GRE sequence was not completed or if there was a patient related factor that degraded one or both imaging sequences. The study was approved by our local Institutional Review Board.

### Image acquisition

All studies were performed on a 1.5 T Siemens Aera magnet (Siemens Healthcare, Erlangen, Germany). For both sequences, vectorcardiographic triggering and respiratory navigation was used. For both 3D bSSFP and 3D IR GRE, a high temporal resolution image was used to select the acquisition period during a quiescent period of the cardiac cycle, field of view (FOV) and phase FOV were adjusted to avoid field wrap, voxel sizes were typically 1.2-1.6 mm, slice thickness was within 0.1 mm of the in-plane resolution to achieve isotropic voxel sizes, and shot duration and timing were similar between the two sequences. 3D bSSFP parameters included TE/TR = 1.2/2.8, flip angle = 90°; 3D IR GRE parameters included TE/TR = 1.3/3.3, flip angle = 18°; IT = 260. Both the 3D bSSFP and the 3D IR GRE sequences used had been optimized for use by the magnet manufacturer. For both sequences, respiratory navigation window was set to achieve 40–60 % image acquisition, and was roughly a 3 mm window for infants, 5 mm for smaller children, and 7 mm for adolescents and young adults. 3D bSSFP imaging was performed pre-contrast; 3D IR GRE imaging was performed 5 min after administration of 0.12 mL/kg (0.03 mmol/kg) GT (max 10 mL). Post-processing was performed using Siemens Argus software version VD 13.

### Image analysis

To evaluate vascular imaging for both 3D bSSFP and 3D IR GRE sequences, a Vascular Imaging Quality Score (VIQS) system was developed (Table 1), which focused on key vascular structures for pediatric CMR, and common issues with image quality. For each patient, right coronary artery (RCA), left coronary artery (LCA), pulmonary arteries, pulmonary veins, and blood pool homogeneity were evaluated with a subscore from 0 (worst) to 3 (best), for a maximal “all-patient VIQS score” of 15. For patients with intrathoracic metal (including those with sternal wires), a sixth subscore indicating the amount of artifact caused by the metal was added, yielding a maximal “metal VIQS score” of 18. Scoring was performed on axial reconstructions of the images generated by each sequence irrespective of the native imaging plane of acquisition. Image scoring was performed on our institution’s PACS system on high resolution Barco monitors to ensure optimal visualization for fine details. The desire to view all images on the highest quality format did necessitate unblinding of the study ID’s for each patient to the reader. To minimize bias, each study was scored for each VIQS subscore by two independent readers (AT and TS) at different sittings separated by at least 24 h for 3D bSSFP and 3D IR GRE for each patient. Differences in subscores were adjudicated by a third experienced reader (WJP). Expected metal object diameter was determined by using other sequences in the study, as well as the pre-CMR chest radiograph.

**Table 1:** Vascular Imaging Quality Score (VIQS) rubric

	0	1	2	3
Right coronary artery (RCA)	Ostium not seen	RCA ostium clear but rest is not	RCA imaged to acute margin	RCA imaged clearly to the inferior cardiac surface
Left coronary artery (LCA)	Ostium not seen	LCA ostium clear but rest is not	LCA bifurcation clear	LAD seen along anterior interventricular groove; LCx seen in posterior AV groove
Pulmonary arteries (PAs)	Branch PAs not clearly defined	RPA & LPA seen	Able to identify RUPA, RLPA, LUPA, LLPA	Able to identify branches off of at least 2 of RUPA, RLPA, LUPA, LLPA
Pulmonary veins	No vein insertion to LA identifiable	2 or more vein insertions clear	All 4 vein insertions clear	All 4 veins clearly seen branching
Blood pool homogeneity	Heterogeneity in a majority of structures	Heterogeneity in multiple chambers/vessels (e.g. LA and LV)	Heterogeneity in single chamber/vessel (e.g. LA)	Homogeneous blood pool
Metal artifact	Artifact >300 % of expected metal object diameter	Artifact >200 % of expected metal object diameter	Artifact >110 % of expected metal object diameter, or asymmetric	Artifact ≤110 % of expected metal object diameter

Shown is the VIQS scoring rubric. For each vascular structure, the sequence must meet all criteria for lower score to achieve a higher score. The RCA, LCA, PA, pulmonary vein, and blood pool homogeneity scores comprise the all-patient VIQS score; the metal artifact score is added for the metal VIQS score

For quantitative analysis, signal- and contrast-to-noise (SNR and CNR) calculations were performed using Syngo MultiModality Workplace (Syngo MMWP) version VE40A (Siemens Healthcare, Erlangen, Germany). Regions of interest were drawn on both the axial 3D bSSFP and 3D IR GRE images in the left ventricular blood pool and myocardium, left atrial blood pool and posterior wall, and proximal right pulmonary artery and vessel wall between the right pulmonary artery and the ascending aorta.

### Statistical analysis

Descriptive statistics were calculated for all variables of interest and included means and standard deviations, and medians and interquartile ranges. Normality of continuous variables was assessed using histograms, normal probability plots, and the Anderson-Darling test for normality. VIQS scores for each subscore were compared using the test of symmetry. Image study duration was compared using paired t-tests. Overall VIQS scores, changes in image quality based on age, and SNR and CNR were compared using Wilcoxon signed-rank testing. Non-metal scores stratified by metal artifact scores and VIQS sores based on age were compared using Kruskal-Wallis testing. Score variability within sequences was compared using Brown-Forsythe testing, and inter-rater agreement was assessed using the weighted kappa statistic. Statistical analyses were performed using SAS 9.4 (Cary, NC) and statistical significance was assessed at the 0.05 level.

## Results

### Patient characteristics

A total of 35 patients completed both 3D bSSFP and 3D IR GRE imaging sequences as part of the same clinical CMR study during the study period, and comprise the all-patient cohort. Of the 35 patients, 17 had intrathoracic metal, and comprise the metal subset cohort. Three other patients were excluded from the study; one for failed ECG gating; one for patient movement, and one for contrast extravasation. Of the 35 patients, 9 underwent the study with general anesthesia and were mechanically ventilated for the CMR, 9 with conscious sedation who were free breathing, and 17 without sedation; the type of sedation used was based on clinical indications. The patients ranged in age from 0.2 to 20.2 years (median 10.7 years, interquartile range (IQR) 5.4–16.1 years). Patient median weight was 46.0 kg (IQR 18.7–63.2 kg). Patient median BSA was 1.43 m^2^ (IQR 0.77–1.65 m^2^). Patients who underwent the studies with general anesthesia had a median age of 4.6 years (IQR 3.1–7.9 years); those with sedation had median age 6.3 years (IQR 5.3–8.4 years), and those who had studies without sedation had median age 16.1 years (IQR 14.8–17.4 years). They represented a broad range of underlying clinical diagnoses, including 11 patients with aortic arch disease including bicuspid aortic valve, aortic dilation/aneurysm, and coarctation; 8 patients with concerns for coronary anomalies; 8 patients with single ventricle anatomy who had undergone Fontan palliation; 5 with repaired tetralogy of Fallot; 3 with inflammatory vasculopathies including Takayasu arteritis or Kawasaki disease; 2 with d-transposition of the great arteries after an arterial switch operation; and one patient with a recurrent left atrial appendage aneurysm (some patients had multiple clinical indications).

### Sequence comparisons

**Fig. 1:**
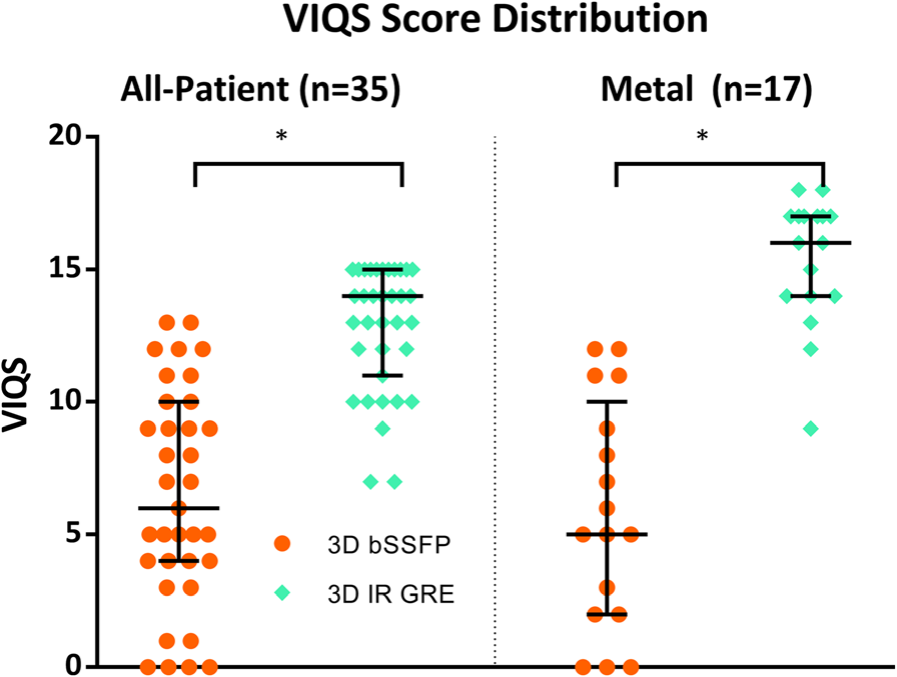
Overall VIQS score distribution. Shown are the VIQS scores (median, interquartile range) for the all-patient VIQS scores for all patients, and metal VIQS scores for the metal cohort, for both 3D bSSFP and 3D IRE GRE imaging. All-patient and metal subset VIQS scores were higher for 3D IR GRE imaging compared to 3D bSSFP imaging (* indicates *p* < 0.0001)

**Fig. 2:**
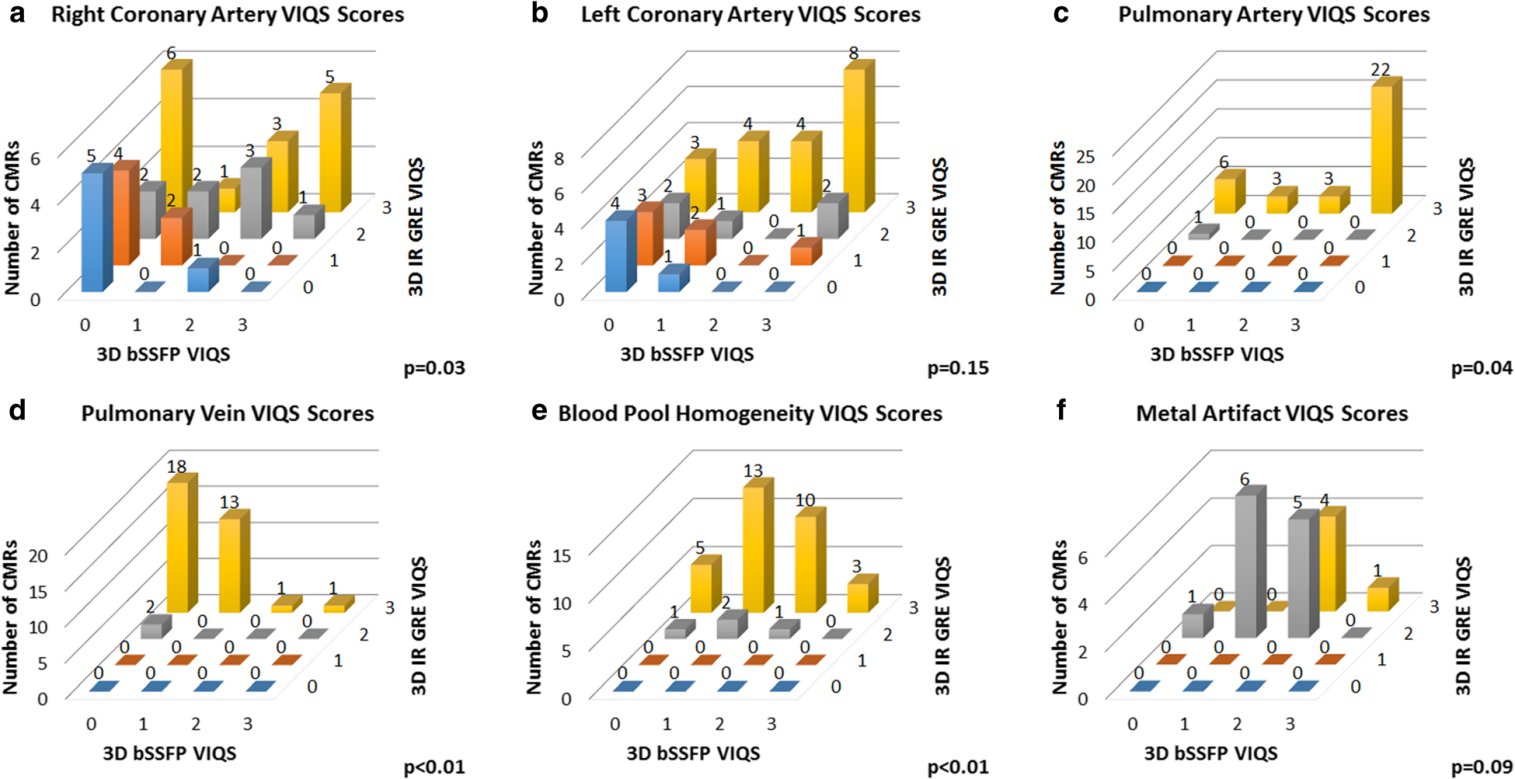
Individual VIQS scores. Shown are the individual VIQS score distributions for 3D bSSFP and 3D IR GRE imaging for (**a**) the right coronary artery; **b** the left coronary artery; **c** the pulmonary arteries; **d** the pulmonary veins; **e** blood pool homogeneity; and (**f**) metal artifact

**Fig. 3:**
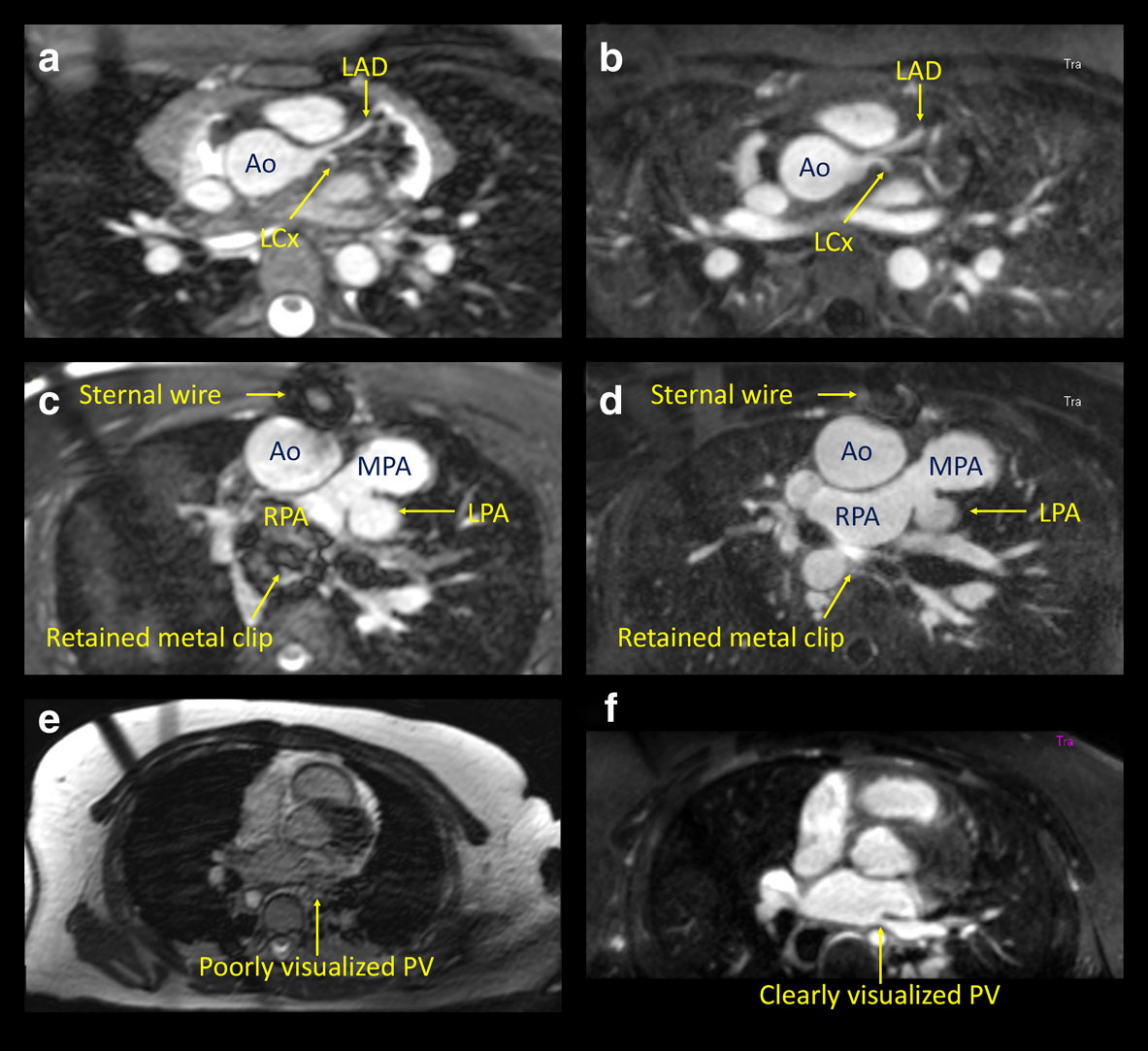
Examples of 3D bSSFP and 3D IR GRE imaging. Excellent left coronary artery imaging by both (**a**) 3D bSSFP and (**b)** 3D IR GRE imaging; a patient with pulmonary atresia status post RV-PA conduit with large metal clip artifact on (**c**) 3D bSSFP, and improved imaging by (**d**) 3D IR GRE; a patient without intrathoracic metal with poorly-visualized left pulmonary vein with (**e**) 3D bSSFP, with improved imaging on 3D IR GRE (**f**)

**Fig. 4:**
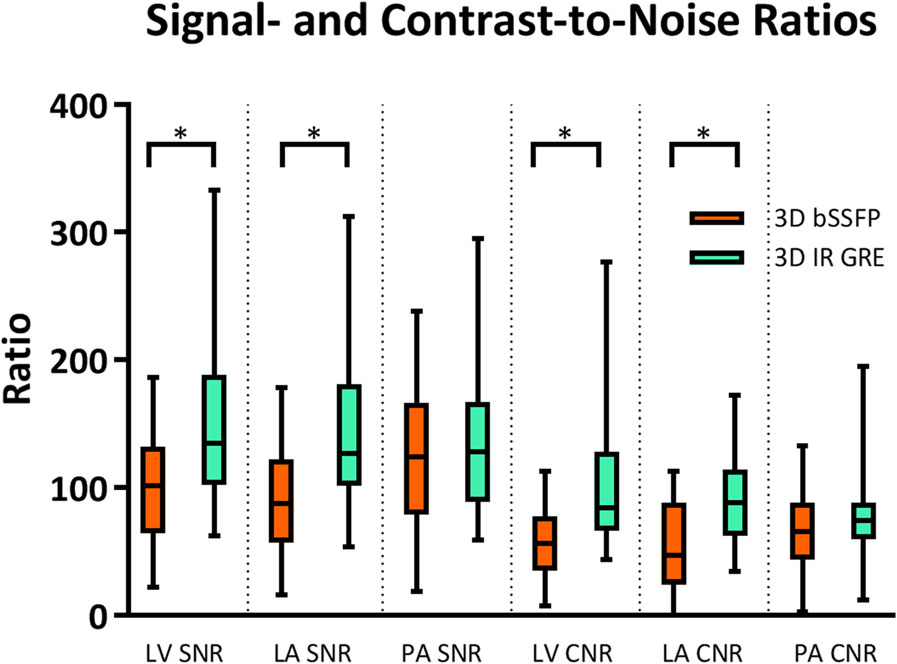
Signal- and contrast-to-noise ratios for 3D bSSFP and 3D IR GRE imaging. Shown are the signal- and contrast-to-noise (SNR and CNR) distributions across the left ventricle, left atrium, and pulmonary arteries (LV, LA, and PA) with bars indicating maximum and minimum values. SNR and CNR ratios were significantly higher in the left ventricle and left atrium with 3D IR GRE imaging as compared to 3D bSSFP imaging (* indicates *p* < 0.0001)

3D IR GRE imaging showed improved overall vascular imaging compared to 3D bSSFP for all patients when comparing all-patient VIQS scores (median 14 (IQR 11–15), vs 6 (4–10), *p* < 0.0001, Fig. 1). When analyzing patients with intrathoracic metal, 3D IR GRE again showed improved VIQS (16 (14–17) vs. 5 (2–9), *p* < 0.0001, Fig. 1). 3D IR GRE showed significantly improved VIQS subscores for imaging the RCA, pulmonary arteries, pulmonary veins, and blood pool homogeneity (Fig. 2 and Table 2). Of the 140 total vascular structures evaluated, only 6 had a lower VIQS score on any of structures on the 3D IR GRE sequence compared to the 3D bSSFP sequence (2 RCA, 4 LCA), while 64 structures (46 %) showed improvement from a 0 or 1 score in the 3D bSSFP to 2 or 3 score on 3D IR GRE (Table 3); this improvement was seen for at least a single vascular structure in 34 of 35 patients. To assess for effects of metal artifact on other scoring, we stratified the non-metal VIQS scores by metal artifact score in the metal cohort, and there was no significant change in the non-metal scores based on the metal score. In addition, 3D IR GRE imaging showed reduced variability in both all-patient and metal VIQS scores compared to 3D bSSFP (both *p* < 0.05). Typical example images are shown in Fig. 3. SNR and CNR were significantly higher for 3D IR GRE compared to 3D bSSFP in the left ventricle and left atrium (*p* < 0.0001), but not the pulmonary arteries (Fig. 4).

**Table 2:** VIQS scores and statistical testing for 3D bSSPF and 3D IR GRE imaging

	3D bSSFP Median (IQR)	3D IR GRE Median (IQR)	*p*-value	3D IR GRE Reduced Variability	Weighted Kappa
RCA	1 (0–2)	2 (1–3)	**0.03** ^**1**^		0.71
LCA	1 (0–3)	3 (1–3)	0.15^1^		0.71
Pulmonary Arteries	3 (1–3)	3 (3–3)	**0.04** ^**1**^		0.66
Pulmonary Veins	0 (0–1)	3 (3–3)	**<0.01** ^**1**^		0.74
Blood Pool Homogeneity	1 (1–2)	3 (3–3)	**<0.01** ^**1**^		0.71
Metal Artifact	2 (1–2)	2 (2–3)	0.09^1^		0.28
Total All-Patient Score	6 (4–10)	14 (11–15)	**<0.0001** ^**2**^	***p*** **= 0.001** ^**3**^	
Total Metal Score (*n* = 17)	5 (2–9)	16 (14–17)	**<0.0001** ^**2**^	***p*** **= 0.043** ^**3**^	

Shown first are median and interquartile ranges for 3D bSSFP and 3D IR GRE imaging VIQS scores for each vascular structure identified. *P*-value column indicates *p*-value of the test of symmetry for the individual VIQS categories, and results of the Wilcoxon signed-rank test for total VIQS scores. 3D IR GRE Reduced Variability column shows results of Brown-Forsythe testing for equality of group variances. Weighted kappa column shows kappa values of agreement for each VIQS category. Bolded *p*-values are less than 0.05

^1^Test of symmetry; ^2^Wilcoxon signed-rank test; ^3^Brown-Forsythe test

**Table 3:** Improvement of VIQS scores from 0 or 1 on 3D bSSFP to 2 or 3 on 3D IR GRE

	*n* = 35
RCA	11 (31 %)
LCA	10 (29 %)
Pulmonary Arteries	10 (29 %)
Pulmonary Veins	33 (94 %)
Blood Pool Homogeneity	21 (60 %)
Metal artifact (*n* = 17)	8 (47 %)

Shown are patients who had a VIQS score of 0 or 1 on 3D bSSFP and 2 or 3 on 3D IR GRE, as a marker of individual VIQS improvements between sequences

3D IR GRE studies had a longer average duration (7:57 ± 4:06 min vs. 6:02 ± 2:26, *p* < 0.01). There was no correlation between heart rate and VIQS for either sequence. Inter-rater agreement assessed by the weighted kappa statistic was considered good on all VIQS subscores except metal artifact (Table 2).

To assess whether the imaging differences were related to age, patients were stratified based on being older or younger than 10 years old. Younger patients had significantly higher 3D IR GRE VIQS scores than 3D bSSFP scores for all but the LCA, whereas older patients had higher VIQS scores for all but RCA, pulmonary arteries, and metal artifacts (Table 4).

**Table 4:** 3D bSSFP vs. 3D IR GRE scores for patients based on age

	Patients < 10 years old (*n* = 16)			Patients ≥ 10 years old (*n* = 19)		
	3D bSSFP Median (IQR)	3D IR GRE Median (IQR)	*p*-value	3D bSSFP Median (IQR)	3D IR GRE Median (IQR)	*p*-value
RCA	0 (0 – 1)	2 (1 – 3)	**0.002**	2 (0 – 3)	3 (1 – 3)	0.113
LCA	1 (0 – 2)	1 (0 – 3)	0.141	2 (0 – 3)	3 (2 – 3)	**0.014**
Pulmonary Arteries	3 (0 – 3)	3 (3 – 3)	**0.008**	3 (2 – 3)	3 (3 – 3)	0.063
Pulmonary Veins	0 (0 – 0)	3 (3 – 3)	**<0.001**	1 (0 – 1)	3 (3 – 3)	**<0.001**
Blood Pool Homogeneity	1 (0 – 2)	3 (3 – 3)	**<0.001**	2 (1 – 2)	3 (3 – 3)	**<0.001**
Metal Artifact	2 (1 – 2)	2 (2 – 2)	**0.031**	2 (1 – 2)	2 (2 – 3)	0.063
Total All-Patient Score	5 (1 – 7)	13 (10 – 14)	**<0.001**	9 (5 – 11)	14 (13 – 15)	**<0.001**
Total Metal Score (*n* = 17)	3 (2 – 7)	16 (14 – 17)	**0.004**	9 (5 – 12)	17 (14 – 17)	**0.008**

Shown are the median (interquartile range) of VIQS scores for each category, based on age of the patient. Bolded *p*-values are less than 0.05

## Discussion

We present a method of vascular CMR, post-GT 3D IR GRE, which we demonstrate to be superior to the standard pre-contrast 3D bSSFP technique in pediatric patients. Since vascular imaging in children remains challenging due to small structure sizes and increased heart rates, uncovering methods of improved vascular imaging is paramount for our field. In addition, delineation of anatomy and size of vascular structures in children is a common indication for CMR, therefore achieving improved vascular imaging is clinically relevant.

We found improved overall imaging with 3D IR GRE in patients with and without metal artifact compared to 3D bSSFP, though the individual metal artifact VIQS subscore was not significantly improved with 3D IR GRE. This finding may be due to our metal artifact VIQS scores having higher inter-rater variability. In some cases, the imaging quality was so poor, particularly for 3D SSFP imaging with intrathoracic metal, that it was difficult to discern the specific causes of image distortion (e.g. whether the poor imaging was due to metal artifact or other reasons). However, given that many patients who are referred for CMR are post-surgical patients with intrathoracic metal, the improved all-patient VIQS with the 3D IR GRE technique was reassuring, even if the differences in the metal artifact subscore were not statistically significant.

We did have a high rate of VIQS scores of 0 for pulmonary veins on the 3D bSSFP imaging. The VIQS scoring system we developed requires the ostium of the vein to be seen clearly entering the left atrium. Given the high rate of signal inhomogeneity/inflow artifacts in the pulmonary veins in 3D bSSFP imaging, this resulted in a number of 0 VIQS scores for pulmonary veins. 3D IR GRE reduces this artifact, therefore improving pulmonary vein VIQS scores (Fig. 3e-f).

There are some limitations to the 3D IR GRE technique. The 3D IR GRE technique did have longer acquisitions on average compared to the 3D bSSFP technique; however, we believe that the improved vascular imaging justifies the approximately 2 min longer sequence duration for this cohort. In addition, the inversion pulse precludes the performance of dual phase imaging due to insufficient time for two inversion pulses in a single RR interval. Though two 3D IR GRE sequences, one in systole and one in diastole, could be run in tandem, we did not investigate this possibility. We also did not investigate the use of different inversion times, as the sequence was used as prescribed by the manufacturer.

Gadofosveset trisodium shows a similar safety profile to standard extracellular gadolinium contrast agents [12], and can be used similarly to extracellular agents for first-pass arterial imaging, though GT offers the advantage of higher relaxivity [13]. Gadofosveset trisodium’s primary advantage is its increased duration of intravascular residence, offering the opportunity to obtain equilibrium images (up to 45–60 min after injection in adults) [13]. GT’s higher relaxivity also allows a smaller total dose of gadolinium to be given (standard dose is 0.03 mmol/kg), and even lower doses of GT have been reported as well [14]. No cases of nephrogenic systemic fibrosis have been reported with GT to date, but given renal clearance, caution should be used in patients with renal impairment, similar to extracellular gadolinium contrast agents [12, 13].

Gadofosveset trisodium’s protein binding and long equilibrium state, however, do confer some limitations to its use. Fibrosis imaging using late gadolinium enhancement (LGE) techniques have not been fully established with GT. While the principals behind LGE imaging hold regardless of the specific gadolinium agent utilized, inversion times and optimal delay between administration and LGE imaging have not been well studied with GT [15]. Additionally, the emerging field of diffuse fibrosis imaging with T1 mapping and extra-cellular volume (ECV) calculation have fundamental assumptions which are violated when using blood pool contrast agents such as GT. Finally, GT is currently unavailable for sale. These limitations notwithstanding, our center had moved toward using GT-enhanced 3D IR GRE imaging for many of our pediatric CMR cases due to its improved vascular imaging in our population, and will do so again when GT is available.

### Limitations

This study does have limitations. We did not randomize the patients to be included in the study. Patient conditions may have been slightly different for the pre-contrast 3D bSSFP and the post-contrast 3D IR GRE, and due to the nature of the sequences, we were unable to alter the fact that the 3D bSSFP had to be run first. Finally, though we did rate the 3D bSSFP and 3D IR GRE datasets in different sittings, there may have been some bias introduced by evaluation of the 3D IR GRE images after the 3D bSSFP.

## Conclusion

In this study, we demonstrate improved imaging of pediatric vascular structures using the post-GT 3D IR GRE technique compared to the pre-contrast 3D bSSFP technique. We recommend its use for many common indications for pediatric CMR. The kinetics of GT do preclude its use for some applications, such as ECV calculation with T1 mapping, but if high quality cardiac and vascular anatomy is the primary clinical question for the study, GT-enhanced 3D IR GRE imaging should be considered.

## Abbreviations

3D bSSFP: Pre-contrast 3D, respiratory navigated, T2 prepared, fat saturated imaging with a balanced steady-state free precession readout
3D IR GRE: 3D, Respiratory navigated, inversion recovery prepared imaging with a gradient echo readout
CMR: Cardiovascular magnetic resonance
CT: Computed tomography
FOV: Field of view
GT: Gadofosveset trisodium
IQR: Interquartile range
LCA: Left coronary artery
LGE: Late gadolinium enhancement
MRA: MRI angiography
MRI: Magnetic resonance imaging
RCA: Right coronary artery
VIQS: Vascular imaging quality score
